# Supplementary material for: Autonomous artificial intelligence increases screening and follow-up for diabetic retinopathy in youth: the ACCESS randomized control trial
Source: Nat Commun. 2024 Jan 11;15:421. doi: 10.1038/s41467-023-44676-z (PMC10784572; doi:10.1038/s41467-023-44676-z)
Supplement: Supplementary file 1 — Supplementary Information [file 41467_2023_44676_MOESM1_ESM.docx]

Autonomous Artificial Intelligence closes the diabetic eye disease care gap in underserved and minority youth: the ACCESS randomized control trial

Supplementary Information

**Table of Contents**

Supplementary Note 1:

- Data Collection Variables page 3

Supplementary Note 2:

- Control arm educational handout page 4

Supplementary Note 3:

- Autonomous AI system, procedure page 5

Supplementary Note 4:

- Autonomous AI result, educational handout page 7

Supplementary Note 5:

- Participant survey page 8

Supplementary Note 6:

- Sample size calculation and Data Governance page 10

Supplementary Note 7:

- Systematic Literature Review on AI and outcomes page 11

Supplementary Note 8:

- Supplementary Tables page 12

## Supplementary Note 1

Data collection

Data were collected from the electronic health record, specifically age, date of birth, sex at birth, race, ethnicity, type of diabetes, date of diabetes diagnosis, medication use (insulin, metformin, GLP1 agonist, etc.), form of insulin administration, use of continuous glucose monitor (CGM) and CGM data, blood pressure, height, weight, body mass index (BMI), presence of other diabetes-related complications (hyperlipidemia, hypertension, macroalbuminuria), abnormal thyroid function, past four hemoglobin A1C readings (if available), diabetic eye exam history, medical history, family medical history, health insurance, and zip code. Parental education status and household income were self- reported by participants using a paper/pencil form.

## Supplementary Note 2

# ACCESS Study

Thank you for participating in the ACCESS Study.

You have been randomized to: **The Standard of Care Group** (which is referral to an eye doctor for the diabetic eye exam).

Risks: Diabetes can lead to complications in the eyes, called diabetic retinopathy, which can cause vision problems or blindness.

Diabetic eye exam: You are being referred for a diabetic eye exam, which is a dilated eye exam to look for diabetes-related eye changes. This can be done at an optometrist, ophthalmologist, or any local eye doctor. Please schedule your diabetic eye exam.

Please send us results: Please ask your eye doctor to fax your diabetic eye exam results to your diabetes provider at:

## Supplementary Note 3

Autonomous AI system

The autonomous AI system (IDx-DR, Digital Diagnostics, Coralville, Iowa, USA) for diagnosing diabetic eye disease (DED) was US FDA De Novo authorized (“FDA approval”) in 2018 for adults with diabetes.^15^ The system diagnoses specific levels of diabetic retinopathy and diabetic macular edema (Early Treatment of Diabetic Retinopathy Study level 35 and higher, clinically significant macular edema, and or center-involved macular edema),^32,33^ referred to as “referable DED,”^34^ that requires further management or treatment by an ophthalmologist or retina specialist. If the ETDRS level is 20 or lower and no macular edema is present, appropriate management is to retest in 12 months.^35^ With this autonomous AI system, a medical diagnosis is made independently by the system without human oversight. In a pivotal trial in a representative sample of adults with diabetes at risk for DED, the safety, efficacy, and lack of racial, ethnic and gender bias of the system was validated against a prognostic standard, i.e. a proxy for patient outcome,^1^ and showed 87% sensitivity and 91% specificity in detecting DED in adults.^2-4^ Since the De Novo authorization of this device,^1^ several other AI systems have become available by demonstrating equivalence to this predicate device.^5,6^ There is variability in the measured sensitivities and specificities of the different AI systems available,^7^ primarily dependent on the quality of the reference standard, with a prognostic standard that includes diabetic macular edema being the most rigorous, ^8,9^ but nevertheless their diagnostic accuracy is higher than clinical experts for detecting DED.^1,10-12^ The IDx-DR autonomous AI is not labeled for youth <22 years, as currently no autonomous AI for DED has been cleared for a pediatric population. In a prospective pilot study in youth with diabetes, we found that thus autonomous AI showed 85.7% sensitivity and 79.3% specificity for the labelled level of DED, ^13^and we therefore applied for and received Investigational Device Exemption from JHH IRB for investigational use in youth. To ensure no cases of disease would be missed, all images were also overread by a board certified retina specialist (TYAL).

Whereas in adults, the recommended protocol for autonomous AI is reflexive pharmacologic dilation, in our pilot studies we found that pharmacologic dilation is unnecessary in youth.^13^ Therefore, in this study, the participant’s eyes were *not* pharmacologically dilated. The autonomous AI system guided the operator to acquire two color fundus images determined to be of adequate quality using an image quality algorithm,^36^ one each centered on the fovea and the optic nerve, and guided the operator to

retake any images of insufficient quality. This process requires approximately 10 minutes, after which the autonomous AI system reports one of the following within 60 seconds: “DED present, refer to specialist”, “DED not present, test again in 12 months”, or “insufficient image quality”. The latter response occurs when the operator is unable to obtain images of adequate quality after 3 attempts.

## Supplementary Note 4

# ACCESS Study

Thank you for participating in the ACCESS Study. You have been randomized to: **The AI Group**

Your diabetic eye exam results today are:

- Normal, no diabetic retinopathy
- Abnormal, more than mild diabetic retinopathy – please go for further evaluation at an eye care provider (optometrist or ophthalmologist).

Please send us results: Please ask your eye doctor to fax your diabetic eye exam results to your diabetes provider at:

## Supplementary Note 5

**Participant Survey**

**Thank you for participating in the ACCESS study, please complete the following questions with you and your caregiver.**

| PARTICIPANT | **Strongly Agree** | **Agree** | **No Opinion/**  **Don’t Know** | **Disagree** | **Strongly Disagree** |
| --- | --- | --- | --- | --- | --- |
| 1. My eyes are healthy. | 1 | 2 | 3 | 4 | 5 |
| 2. I know diabetes could have an impact on my eyesight. | 1 | 2 | 3 | 4 | 5 |
| 3. Having a diabetic eye exam regularly is important. | 1 | 2 | 3 | 4 | 5 |
| EYE EXAM | **Very Satisfied** | **Satisfied** | **No Opinion –**  **Neither satisfied nor dissatisfied** | **Not Satisfied** | **Very Not Satisfied** |
| 4. How satisfied were you with the length of time it took to complete the diabetic eye exam? | 1 | 2 | 3 | 4 | 5 |
| 5. How satisfied were you with the length of time it took to *receive the results* of your diabetic eye exam? | 1 | 2 | 3 | 4 | 5 |
| 6. How satisfied were you that you received an easy to understand explanation of procedures before the eye exam? | 1 | 2 | 3 | 4 | 5 |
| 7. If applicable, how satisfied were you with the overall experience of having a diabetic eye exam done in clinic? | 1 | 2 | 3 | 4 | 5 |
| **PLANNING FOR YOUR NEXT DIABETIC EYE EXAM** | **Very Likely** | **Likely** | **No Opinion –**  **Neither likely nor not likely** | **Not Likely** | **Not Very Likely** |

| 8. How likely are you to choose a **dilated eye exam** at an eye care provider for your next diabetic eye exam? | 1 | 2 | 3 | 4 | 5 |
| --- | --- | --- | --- | --- | --- |
| 9. How likely are you to choose a **point of care diabetic retinopathy screening exam using artificial intelligence** for your next diabetic eye exam? | 1 | 2 | 3 | 4 | 5 |

## Supplementary Note 6

Sample Size Calculation

We assumed that a 20% difference in DED screening completion rates (care gap closure) would be clinically relevant. We calculated that a sample size of 164 (n=82, n=82 AI) would provide 80% power with a 2-tailed type-1 error of 0.05. Since randomization and study visit occurred at the same time there was little risk of attrition and thus the sample size was not expanded to account for attrition.

Data Governance

Although this was a low-risk clinical trial, an independent Data Safety and Monitoring Board was established to protect the interests of study participants and to preserve the integrity and credibility of the study data, based on pre-specified aims, thereby reducing any concerns that interim data could influence or bias the study results and interpretation. At the time of the DSMB meeting on 9/16/2022, all participants had already been enrolled in the trial, and the DSMB determined that the study should continue to completion.

## Supplementary Note 7

Systematic literature review

We performed a Clinical Queries Therapy/Broad PubMed search in December 2022, using MESH terms: ((artificial intelligence" OR "AI" OR "clinical decision support [MeSH Major Topic]) AND (("outcome" OR "care gap" OR "compliance" OR "health equity" OR "health disparity" [MeSH Major Topic])) AND (("clinical"[Title/Abstract] AND "trial"[Title/Abstract]) OR "clinical trials as topic"[MeSH Terms] OR "clinical trial"[Publication Type] OR "random*"[Title/Abstract] OR "random allocation"[MeSH Terms] OR "therapeutic use"[MeSH Subheading]). This search was performed by 3 independent individuals (RC, HPL, MDA), which results in 4 articles, with no published articles of diagnostic autonomous AI in closing a guideline-based care gap.

1. Artificial intelligence-enabled electrocardiograms for identification of patients with low ejection fraction: a pragmatic, randomized clinical trial.

Yao X, Rushlow DR, Inselman JW, McCoy RG, Thacher TD, Behnken EM, Bernard ME, Rosas SL, Akfaly A, Misra A, Molling PE, Krien JS, Foss RM, Barry BA, Siontis KC, Kapa S, Pellikka PA, Lopez-Jimenez F, Attia ZI, Shah ND, Friedman PA, Noseworthy PA.

Nat Med. 2021 May;27(5):815-819. doi: 10.1038/s41591-021-01335-4. Epub 2021 May 6.

1. Insulin dose optimization using an automated artificial intelligence-based decision support system in youths with type 1 diabetes.

Nimri R, Battelino T, Laffel LM, Slover RH, Schatz D, Weinzimer SA, Dovc K, Danne T, Phillip M; NextDREAM Consortium.

Nat Med. 2020 Sep;26(9):1380-1384. doi: 10.1038/s41591-020-1045-7. Epub 2020 Sep 9.

1. Rho MJ, Park J, Moon HW, Lee C, Nam S, Kim D, Kim CS, Jeon SS, Kang M, Lee JY. Dr. Answer AI for prostate cancer: Clinical outcome prediction model and service. PLoS One. 2020 Aug 5;15(8):e0236553. doi: 10.1371/journal.pone.0236553. PMID: 32756597; PMCID: PMC7406030.
2. Simon G, DiNardo CD, Takahashi K, Cascone T, Powers C, Stevens R, Allen J, Antonoff MB, Gomez D, Keane P, Suarez Saiz F, Nguyen Q, Roarty E, Pierce S, Zhang J, Hardeman Barnhill E, Lakhani K, Shaw K, Smith B, Swisher S, High R, Futreal PA, Heymach J, Chin L. Applying Artificial Intelligence to Address the Knowledge Gaps in Cancer Care. Oncologist. 2019 Jun;24(6):772-782. doi: 10.1634/theoncologist.2018-0257. Epub 2018 Nov 16. PMID: 30446581; PMCID: PMC6656515.

## Supplementary Note 8

**Table S1: ACCESS Patient Characteristics by Location**

| Factor | All | JHU | MWPH | p-value |
| --- | --- | --- | --- | --- |
| N | 164 | 82 | 82 |  |
| Age, mean (SD) | 15.2 (2.8) | 14.8 (2.5) | 15.6 (3.0) | 0.057 |
| Race |  |  |  | <0.001 |
| Asian | 10 (6.1%) | 3 (3.7%) | 7 (8.5%) |  |
| NH Black | 58 (35.4%) | 40 (48.8%) | 18 (22.0%) |  |
| Hispanic | 10 (6.1%) | 8 (9.8%) | 2 (2.4%) |  |
| NH White | 86 (52.4%) | 31 (37.8%) | 55 (67.1%) |  |
| Male Sex | 68 (41.5%) | 30 (36.6%) | 38 (46.3%) | 0.20 |
| Household Income |  |  |  | 0.44 |
| $25,000 or less | 25 (15.2%) | 16 (19.5%) | 9 (11.0%) |  |
| $25,000-$49,999 | 31 (18.9%) | 17 (20.7%) | 14 (17.1%) |  |
| $50,000-$74,999 | 28 (17.1%) | 14 (17.1%) | 14 (17.1%) |  |
| $75,000-$99,999 | 16 (9.8%) | 6 (7.3%) | 10 (12.2%) |  |
| More than $100,000 | 48 (29.3%) | 20 (24.4%) | 28 (34.1%) |  |
| Choose not to answer/refused | 16 (9.8%) | 9 (11.0%) | 7 (8.5%) |  |
| Highest Education |  |  |  | 0.005 |
| Less than 12 years of High School | 5 (3.0%) | 5 (6.1%) | 0 (0.0%) |  |
| High School/GED | 57 (34.8%) | 30 (36.6%) | 27 (32.9%) |  |
| Associate’s Degree | 19 (11.6%) | 7 (8.5%) | 12 (14.6%) |  |
| Undergraduate Degree | 25 (15.2%) | 8 (9.8%) | 17 (20.7%) |  |
| Post-Graduate Degree | 51 (31.1%) | 25 (30.5%) | 26 (31.7%) |  |
| Unknown | 7 (4.3%) | 7 (8.5%) | 0 (0.0%) |  |
| Medicaid | 77 (47.0%) | 45 (54.9%) | 32 (39.0%) | 0.042 |
| Type 1 Diabetes | 119 (72.6%) | 47 (57.3%) | 72 (87.8%) | <0.001 |
| Type 2 Diabetes | 45 (27.4%) | 35 (42.7%) | 10 (12.2%) | <0.001 |
| Duration of diabetes (years), median (IQR) | 5.8 (3.2, 8.7) | 4.0 (1.3, 7.3) | 8.3 (5.3, 10.1) | <0.001 |
| HbA1C value at this visit, mean (SD) | 8.6 (2.3) | 8.5 (2.5) | 8.6 (1.9) | 0.76 |
| Continuous glucose monitor use | 125 (76.2%) | 57 (69.5%) | 68 (82.9%) | 0.044 |
| Has ever had prior diabetic eye exam | 129 (78.7%) | 57 (69.5%) | 72 (87.8%) | 0.004 |

* p-values were calculated using Chi-Squared tests for categorical variables, Wilcoxon rank-sum test for duration of diabetes and Student’s t tests for all other continuous variables. All statistical tests two-sided.

**Table S2: Sensitivity analysis excluding 3 participants lost to followup in the control arm (n=160)**

| Table S2a. Primary Outcome Sensitivity analysis | | | | | | | | | |
| --- | --- | --- | --- | --- | --- | --- | --- | --- | --- |
| Factor | | Value | | Did not have screening | | Had screening | | p-value | |
| N (%) | | 160 | | 61 (38.1%) | | 99 (61.9%) | | <.001 | |
| Age, mean (SD) | | 15.2 (2.8) | | 14.8 (2.8) | | 15.4 (2.8) | | 0.16 | |
| Race | |  | |  | |  | |  | |
| Asian | | 10 (6.2%) | | 3 (4.9%) | | 7 (7.1%) | | 0.58 | |
| Black | | 57 (35.6%) | | 21 (34.4%) | | 36 (36.4%) | | 0.80 | |
| Hispanic | | 10 (6.2%) | | 4 (6.6%) | | 6 (6.1%) | | 0.90 | |
| White | | 83 (51.9%) | | 33 (54.1%) | | 50 (50.5%) | | 0.66 | |
| Male Sex | | 67 (41.9%) | | 29 (47.5%) | | 38 (38.4%) | | 0.25 | |
| Household Income | |  | |  | |  | | 0.82 | |
| <$50,000 per year | | 55 (34.4%) | | 22 (36.1%) | | 33 (33.3%) | |  | |
| >= $50,000 per year | | 89 (55.6%) | | 34 (55.7%) | | 55 (55.6%) | |  | |
| Unknown | | 16 (10.0%) | | 5 (8.2%) | | 11 (11.1%) | |  | |
| Highest Education | |  | |  | |  | | 0.49 | |
| High School or less | | 60 (37.5%) | | 20 (32.8%) | | 40 (40.4%) | |  | |
| More than High School | | 93 (58.1%) | | 39 (63.9%) | | 54 (54.5%) | |  | |
| Unknown | | 7 (4.4%) | | 2 (3.3%) | | 5 (5.1%) | |  | |
| Medicaid insurance | | 75 (46.9%) | | 30 (49.2%) | | 45 (45.5%) | | 0.65 | |
| Type 1 Diabetes | | 117 (73.1%) | | 46 (75.4%) | | 71 (71.7%) | | 0.61 | |
| Type 2 Diabetes | | 43 (26.9%) | | 15 (24.6%) | | 28 (28.3%) | | 0.61 | |
| Duration of diabetes (years), median (IQR) | | 5.8 (3.3, 8.7) | | 6.3 (3.3, 9.2) | | 5.3 (3.3, 8.4) | | 0.40 | |
| HbA1c value at study visit, mean (SD) | | 8.6 (2.2) | | 8.6 (2.2) | | 8.6 (2.3) | | 1.00 | |
| Continuous glucose monitor use | | 123 (76.9%) | | 48 (78.7%) | | 75 (75.8%) | | 0.67 | |
|  | |  | |  | |  | |  |  |
|  | |  | |  | |  | |  |  |
|  | |  | |  | |  | |  |  |

Table S2b. Secondary Outcome Sensitivity analysis - excluding 3 patients lost to follow up (all SOC)

|  | Control | AI | Total | p-value |
| --- | --- | --- | --- | --- |
| No follow up dilated exam, n (%) | 61 (77%) | 9 (36%) | 70 (67%) | <.0001 |
| Yes, follow up dilated exam, n (%) | 18 (23%) | 16 (64%) | 34 (33%) |  |

* p-values were calculated using Chi-Squared tests for categorical variables, Wilcoxon rank-sum test for duration of diabetes and Student’s t tests for all other continuous variables. All statistical tests two-sided.

**Table S3: Sensitivity analysis classifying 3 participants lost to follow up as screening complete in the control arm (n=163)**

Table S3a. Primary Outcome Sensitivity analysis

| Factor | Value | Did not have screening | Had screening | p-value |
| --- | --- | --- | --- | --- |
| N | 163 | 61 (37.4%) | 102 (62.6%) | <.001 |
| Age, mean (SD) | 15.2 (2.8) | 14.8 (2.8) | 15.5 (2.8) | 0.13 |
| Race |  |  |  |  |
| Asian | 10 (6.1%) | 3 (4.9%) | 7 (6.9%) | 0.62 |
| Black | 58 (35.6%) | 21 (34.4%) | 37 (36.3%) | 0.81 |
| Hispanic | 10 (6.1%) | 4 (6.6%) | 6 (5.9%) | 0.86 |
| White | 85 (52.1%) | 33 (54.1%) | 52 (51.0%) | 0.7 |
| Male Sex | 68 (41.7%) | 29 (47.5%) | 39 (38.2%) | 0.24 |
| Household Income |  |  |  | 0.84 |
| <$50,000 per year | 56 (34.4%) | 22 (36.1%) | 34 (33.3%) |  |
| >= $50,000 per year | 91 (55.8%) | 34 (55.7%) | 57 (55.9%) |  |
| Unknown | 16 (9.8%) | 5 (8.2%) | 11 (10.8%) |  |
| Highest Education |  |  |  | 0.45 |
| High School or less | 62 (38.0%) | 20 (32.8%) | 42 (41.2%) |  |
| More than High School | 94 (57.7%) | 39 (63.9%) | 55 (53.9%) |  |
| Unknown | 7 (4.3%) | 2 (3.3%) | 5 (4.9%) |  |
| Medicaid insurance | 77 (47.2%) | 30 (49.2%) | 47 (46.1%) | 0.7 |
| Type 1 Diabetes | 118 (72.4%) | 46 (75.4%) | 72 (70.6%) | 0.51 |
| Type 2 Diabetes | 45 (27.6%) | 15 (24.6%) | 30 (29.4%) | 0.51 |
| Duration of diabetes (years), median (IQR) | 5.8 (3.2, 8.7) | 6.3 (3.3, 9.2) | 5.3 (3.2, 8.4) | 0.35 |
| HbA1c value at this visit, mean (SD) | 8.6 (2.2) | 8.6 (2.2) | 8.5 (2.3) | 0.95 |
| Continuous glucose monitor use | 124 (76.1%) | 48 (78.7%) | 76 (74.5%) | 0.55 |

Table S3b. Secondary outcome Sensitivity analysis - classifying 3 patients lost to follow up as screened (all SOC)

|  | Control | AI | Total | p-value |
| --- | --- | --- | --- | --- |
| No, follow up dilated exam, n (%) | 61 (74%) | 9 (36%) | 70 (65%) | <.0001 |
| Yes, follow up dilated exam, n (%) | 21 (26%) | 16 (64%) | 37 (35%) |  |

* p-values were calculated using Chi-Squared tests for categorical variables, Wilcoxon rank-sum test for duration of diabetes and Student’s t tests for all other continuous variables. All statistical tests two-sided.

References

1. Abramoff MD, Lavin PT, Birch M, Shah N, Folk JC. Pivotal trial of an autonomous AI-­‐based diagnostic system for detection of diabetic retinopathy in primary care offices. NPJ Digit Med 2018;1:39.
2. Lin DY, Blumenkranz MS, Brothers RJ, Grosvenor DM. The sensitivity and specificity of single-­‐field nonmydriatic monochromatic digital fundus photography with remote image interpretation for diabetic retinopathy screening: a comparison with ophthalmoscopy and standardized mydriatic color photography. Am J Ophthalmol 2002;134:204-­‐13.
3. Liu Y, Rajamanickam VP, Parikh RS, et al. Diabetic Retinopathy Assessment Variability Among Eye Care Providers in an Urban Teleophthalmology Program. Telemed J E Health 2019;25:301-­‐8.
4. Pugh JA, Jacobson JM, Van Heuven WA, et al. Screening for diabetic retinopathy. The wide-­‐angle retinal camera. Diabetes Care 1993;16:889-­‐95.
5. Ipp E, Liljenquist D, Bode B, et al. Pivotal Evaluation of an Artificial Intelligence System for Autonomous Detection of Referrable and Vision-­‐Threatening Diabetic Retinopathy. JAMA network open 2021;4:e2134254.
6. CISION. AEYE Health receives FDA clearance for AI-­‐based autonomous screening for referable diabetic retinopathy. 2022.
7. Lee AY, Yanagihara RT, Lee CS, et al. Multicenter, Head-­‐to-­‐Head, Real-­‐World Validation Study of Seven Automated Artificial Intelligence Diabetic Retinopathy Screening Systems. Diabetes Care 2021;44:1168-­‐75.
8. Abràmoff MD, Cunningham B, Patel B, et al. Foundational Considerations for Artificial Intelligence Using Ophthalmic Images. Ophthalmology 2022;129:e14-­‐e32.
9. Abràmoff MD, Tobey D, Char DS. Lessons Learned About Autonomous AI: Finding a Safe, Efficacious, and Ethical Path Through the Development Process. Am J Ophthalmol 2020;214:134-­‐42.
10. Verbraak FD, Abramoff MD, Bausch GCF, et al. Diagnostic Accuracy of a Device for the Automated Detection of Diabetic Retinopathy in a Primary Care Setting. Diabetes Care 2019;42:651-­‐6.
11. Ting DSW, Cheung CY, Lim G, et al. Development and Validation of a Deep Learning System for Diabetic Retinopathy and Related Eye Diseases Using Retinal Images From Multiethnic Populations With Diabetes. Jama 2017;318:2211-­‐23.
12. Gulshan V, Peng L, Coram M, et al. Development and Validation of a Deep Learning Algorithm for Detection of Diabetic Retinopathy in Retinal Fundus Photographs. JAMA 2016;316:2402-­‐10.
13. Wolf RM, Liu TYA, Thomas C, et al. The SEE Study: Safety, Efficacy, and Equity of Implementing Autonomous Artificial Intelligence for Diagnosing Diabetic Retinopathy in Youth. Diabetes Care 2021;44:781-­‐7.
