# Supplementary material for: Autonomous artificial intelligence increases screening and follow-up for diabetic retinopathy in youth: the ACCESS randomized control trial
Source: Nat Commun. 2024 Jan 11;15:421. doi: 10.1038/s41467-023-44676-z (PMC10784572; doi:10.1038/s41467-023-44676-z)
Supplement: Supplementary file 3 — Reporting Summary [file 41467_2023_44676_MOESM3_ESM.pdf]

nature portfolio

Corresponding author(s): Risa Wolf, MD

Last updated by author(s): 12/17/2023

Reporting Summary

Nature Portfolio wishes to improve the reproducibility of the work that we publish. This form provides structure for consistency and transparency in reporting. For further information on Nature Portfolio policies, see our Editorial Policies and the Editorial Policy Checklist.

Statistics

For all statistical analyses, confirm that the following items are present in the figure legend, table legend, main text, or Methods section.

n/a	Confirmed	
☐	☒	The exact sample size (*n*) for each experimental group/condition, given as a discrete number and unit of measurement
☐	☒	A statement on whether measurements were taken from distinct samples or whether the same sample was measured repeatedly
☐	☒	The statistical test(s) used AND whether they are one- or two-sided<br>*Only common tests should be described solely by name; describe more complex techniques in the Methods section.*
☐	☒	A description of all covariates tested
☐	☒	A description of any assumptions or corrections, such as tests of normality and adjustment for multiple comparisons
☐	☒	A full description of the statistical parameters including central tendency (e.g. means) or other basic estimates (e.g. regression coefficient) AND variation (e.g. standard deviation) or associated estimates of uncertainty (e.g. confidence intervals)
☐	☒	For null hypothesis testing, the test statistic (e.g. *F*, *t*, *r*) with confidence intervals, effect sizes, degrees of freedom and *P* value noted<br>*Give P values as exact values whenever suitable.*
☒	☐	For Bayesian analysis, information on the choice of priors and Markov chain Monte Carlo settings
☒	☐	For hierarchical and complex designs, identification of the appropriate level for tests and full reporting of outcomes
☒	☐	Estimates of effect sizes (e.g. Cohen's *d*, Pearson's *r*), indicating how they were calculated

Our web collection on statistics for biologists contains articles on many of the points above.

Software and code

Policy information about availability of computer code

Data collection	Redcap
Data analysis	Statacorp 15.1

For manuscripts utilizing custom algorithms or software that are central to the research but not yet described in published literature, software must be made available to editors and reviewers. We strongly encourage code deposition in a community repository (e.g. GitHub). See the Nature Portfolio guidelines for submitting code & software for further information.

Data

Policy information about availability of data

All manuscripts must include a data availability statement. This statement should provide the following information, where applicable:

- Accession codes, unique identifiers, or web links for publicly available datasets
- A description of any restrictions on data availability
- For clinical datasets or third party data, please ensure that the statement adheres to our policy

Data from this study will be shared with bona fide researchers submitting a research proposal approved by the primary investigator. Data will be shared in a de-identified/anonymized format. The controlled access is implemented to safeguard the confidentiality and sensitive nature of the data. For access requests, please contact Risa Wolf at rwolf@jhu.edu. Access to the data will be granted upon review of the request, and researchers can expect a response within 90 days from the date of submission. Access to the data is contingent upon the completion of a data use agreement (DUA). The DUA outlines the terms and conditions for data usage, including restrictions on data sharing, publication, and any other pertinent considerations. Researchers granted access will be required to abide by the terms

specified in the DUA to ensure the responsible and ethical use of the data.

Research involving human participants, their data, or biological material

Policy information about studies with human participants or human data. See also policy information about sex, gender (identity/presentation), and sexual orientation and race, ethnicity and racism.

Reporting on sex and gender	Yes
Reporting on race, ethnicity, or other socially relevant groupings	Yes
Population characteristics	Youth with T1D (11-21 years) or T2D (8-21 years) were eligible for inclusion if they met criteria for DED screening per American Diabetes Association (ADA) 2021 guidelines,16 had no known DED, and had not had a diabetic eye exam within the last 6 months.
Recruitment	Potential study participants were approached in the diabetes clinic to confirm eligibility and then recruited by a study coordinator with written informed consent.
Ethics oversight	The study was approved by the Johns Hopkins IRB, the tenets of th e Declaration of Helsinki were followed, and an independent Data Safety and Monitoring Board was established. Potential study participants were approached in the diabetes clinic to confirm eligibility and then recruited by a study coordinator with written informed consent.

Note that full information on the approval of the study protocol must also be provided in the manuscript.

Field-specific reporting

Please select the one below that is the best fit for your research. If you are not sure, read the appropriate sections before making your selection.

☐ Life sciences ☒ Behavioural & social sciences ☐ Ecological, evolutionary & environmental sciences

For a reference copy of the document with all sections, see nature.com/documents/nr-reporting-summary-flat.pdf

Behavioural & social sciences study design

All studies must disclose on these points even when the disclosure is negative.

Study description	ACCESS is a hypothesis-driven, pre-registered, prospective parallel, RCT with a 1:1 allocation ratio that was conducted at the Johns Hopkins Pediatric Diabetes Center at two sites (Johns Hopkins Hospital and Mount Washington Pediatric Hospital) in Baltimore, Maryland, which serves a racially and ethnically diverse population.
Research sample	164 participants completed informed consent and were randomized. Baseline characteristics were similar in the two groups (Table 1): mean age 15.2y (SD 2.8), 58% female, 35% Black, 6% Hispanic, 47% Medicaid insurance, 34% with household income <$50,000, 38% with parental education of up to high school completion, 73% had T1D, median duration of diabetes was 5.8 years, and mean HbA1c was 8.6%.
Sampling strategy	The sampling strategy was based on a convenience sample of youth seen for diabetes care in the two recruitment sites. If eligible, participants were consented and then randomized.<br>We assumed that a 20% difference in DED screening completion rates (care gap closure) would be clinically relevant. Based on our prior study,30 where baseline screening rates before AI were 49%, we assumed that with the educational intervention, screening rates for usual care would be closer to 60% in this study, and demonstrating a difference of 20% would be clinically relevant for AI screening. We calculated that a sample size of 164 (n=82, n=82 AI) would provide 80% power with a 2-tailed type-1 error of 0.05. Since randomization and study visit occurred at the same time there was little risk of attrition and thus the sample size was not expanded to account for attrition.
Data collection	Data were collected from the electronic health record, specifically age, date of birth, sex at birth, race, ethnicity, type of diabetes, date of diabetes diagnosis, medication use (insulin, metformin, GLP1 agonist, etc.), form of insulin administration, use of continuous glucose monitor (CGM) and CGM data, blood pressure, height, weight, body mass index (BMI), presence of other diabetes-related complications (hyperlipidemia, hypertension, macroalbuminuria), abnormal thyroid function, past four hemoglobin A1C readings (if available), diabetic eye exam history, medical history, family medical history, health insurance, and zip code. Parental education status and household income were self-reported by participants using a paper/pencil form. The researcher was masked to the experimental conditions until randomization.
Timing	Participants were enrolled from November 24, 2021, through June 6, 2022, and follow-up was completed by Dec 6, 2022.
Data exclusions	There were 3 participants in the control arm who did not return for clinic visits, were not able to be reached by phone or EHR-based messaging, and thus their eye exams were considered incomplete. Sensitivity analysis was performed excluding these 3 participants, and also assuming they completed their eye exams, and the results were unchanged.
Non-participation	Six patients who were approached declined participation.

Randomization	To prevent selection bias and ensure sample size balance between the groups and sites, stratified randomization (by site) was used, and participants were randomized in permutated block schedules of 4 and 6. Within each block, participants were randomized with a 1:1 allocation ratio to the control group and intervention group. This randomization sequence was created by a statistician unaffiliated with the study to ensure masking to the randomization scheme and was implemented by REDCAP's randomization software based on the participant's location.52,53 After consent, the research coordinator entered the participant location and the randomization allocation was generated. All parties were masked to the allocation until the participant was randomized in the study, and then all parties were unmasked.

Reporting for specific materials, systems and methods

We require information from authors about some types of materials, experimental systems and methods used in many studies. Here, indicate whether each material, system or method listed is relevant to your study. If you are not sure if a list item applies to your research, read the appropriate section before selecting a response.

Materials & experimental systems

n/a	Involved in the study	
☒	☐	Antibodies
☒	☐	Eukaryotic cell lines
☒	☐	Palaeontology and archaeology
☒	☐	Animals and other organisms
☐	☒	Clinical data
☒	☐	Dual use research of concern
☒	☐	Plants

Methods

n/a	Involved in the study	
☒	☐	ChIP-seq
☒	☐	Flow cytometry
☒	☐	MRI-based neuroimaging

Clinical data

Policy information about clinical studies

All manuscripts should comply with the ICMJEguidelines for publication of clinical research and a completedCONSORT checklist must be included with all submissions.

Clinical trial registration	NCT05131451
Study protocol	Included as a supplement
Data collection	This study was conducted at the Johns Hopkins Pediatric Diabetes Center at two sites (Johns Hopkins Hospital and Mount Washington Pediatric Hospital) in Baltimore, Maryland. Participants were enrolled from November 24, 2021, through June 6, 2022, and follow-up was completed by Dec 6, 2022.
Outcomes	In order to test the primary hypothesis, the pre-specified primary outcome was defined as the proportion of participants who completed a documented diabetic eye exam in each arm ("primary care gap closure rate"). In the control arm, this is the proportion of patients who completed a documented diabetic eye exam with an ECP within 6 months of randomization; in the intervention arm, this is the proportion of participants who completed the autonomous AI exam at the study visit.
